# Diagnostic approach to limbal stem cell deficiency

**DOI:** 10.3389/fopht.2024.1524595

**Published:** 2025-01-16

**Authors:** Daniella Lent-Schochet, Mizna Akbar, Joshua H. Hou, Asim V. Farooq

**Affiliations:** ^1^ Department of Ophthalmology and Visual Science, University of Chicago, Chicago, IL, United States; ^2^ Department of Ophthalmology and Visual Neurosciences, University of Minnesota, Minnesota, MN, United States

**Keywords:** limbal stem cell deficiency, anterior segment OCT, confocal micoscopy, impression cytology, limbal stem cell

## Abstract

Limbal stem cell deficiency (LSCD) is an important cause of visual and ocular morbidity. Effective diagnosis and management require a thoughtful and comprehensive evaluation of the ocular surface. This review describes the pathogenesis, diagnosis, and grading of LSCD, as well as characteristic findings via slit lamp examination, *in-vivo* confocal microscopy, anterior segment optical coherence tomography (AS-OCT), impression cytology, and OCT angiography.

## 1 Introduction

Limbal stem cell deficiency (LSCD) is a disorder of the ocular surface where corneal epithelial homeostasis is disrupted by dysfunction or an insufficient quantity of limbal stem cells (LSCs). The past few decades have ushered in significant advancements in the detection and treatment of LSCD, and recently the lack of clear diagnostic standards was addressed by the Cornea Society’s Limbal Stem Cell Working Group, which established a consensus amongst international experts on LSCD diagnosis and classification (1). Accurate diagnosis and staging is critical for selecting the treatment that is best suited for a patient’s disease severity (2). Treatments can range from medical management alone to surgery. Kim, et al. described cases of LSC dysfunction where medical therapy alone was sufficient to reverse signs of LSCD (3). Corneal epithelial removal with or without amniotic membrane transplantation, conjunctival limbal autograft, conjunctival limbal allograft, keratolimbal allograft, cultivated limbal epithelial stem cells, simple limbal epithelial transplantation and keratoprosthesis surgery have been described as surgical treatments for cases of LSCD; in unilateral cases, autologous versions of LSC transplant can be considered with harvesting from the contralateral eye (1). Mucous membrane graft can also be considered, especially in the presence of keratinization of the lid margin and palpebral conjunctiva (4). In this review, we summarize the current diagnostic paradigm for LSCD.

## 2 Pathophysiology, etiology, and epidemiology of LSCD

LSCs reside in the limbus, which is a 1-2mm transition zone where corneal and conjunctival epithelia meet (5). The limbus hosts LSCs in the basal region, in fibrovascular ridges called the palisades of Vogt (6). Based on the widely accepted *XYZ hypothesis* of corneal maintenance, these LSCs give rise to transient amplifying cells (TACs) that migrate centripetally along the basal layer of the epithelium. Differentiated offsprings of these TACs then undertake anterior migration and further differentiation into mature corneal epithelium (7). The limbus also plays an important role as the barrier between the avascular cornea and vascular conjunctiva (8). Deficiency of LSCs can lead to dysfunction in corneal epithelial homeostasis, resulting in persistent epithelial defects, neovascularization, ulceration, opacification, and conjunctivalization of the cornea (5).

Causes of LSCD are best divided into acquired and non-acquired. LSCD can be acquired by immune-mediated conditions including Stevens-Johnson Syndrome/toxic necrolysis (SJS/TEN) (9, 10), chronic ocular graft vs host disease (GVHD) (11), atopic/allergic ocular surface disease, and mucous membrane pemphigoid (12). Acquired, non-immune mediated causes include direct damage from radiation, contact lens wear, chemical or thermal burns, drug-induced injury such as systemic anti-neoplastic medications, limbal surgeries, infectious ocular diseases, ocular surface tumors, chronic lid margin disease, severe pterygium, and toxic exposures such as mustard gas (1, 13–19). Traumatic etiologies are more likely to cause asymmetrical LSCD (1). Non-acquired causes include genetic diseases such as congenital aniridia, xeroderma pigmentosum, and dyskeratosis congenita (20–22).

A multi-center study in India including 1331 patients identified ocular surface burns as the most common cause of unilateral LSCD (84%). For bilateral LSCD, the most frequent causes were allergic conjunctivitis (29%), SJS/TEN (23%), and congenital aniridia (9%) (23). Despite LSCD placing a significant burden on selected cornea subspecialty clinics, there are limited epidemiological data for LSCD in the U.S (24). A handful of epidemiological studies have been published to date. Goldberg, et al. found an LSCD prevalence of 4.25% over a two year period at a cornea subspeciality tertiary referral center (24). While the authors note the likely impact of referral bias and underdiagnosis prior to arrival at their tertiary center, this prevalence is significantly higher than the estimated prevalence in the U.S. reported by Orphanet rare disease (0.0001-0.0005%) (24). Cheung et al. conducted a single center study of 434 patients and found that congenital aniridia was the most common cause of LSCD accounting for 31% of cases, followed by chemical or thermal injuries accounting for 21% (25). Cheung also found that 70% of their patients had bilateral disease, and the average patient was middle-aged with no sex predominance (25). Haring, et al. studied epidemiological trends in the U.S. specifically for chemical ocular burns and found that children aged 1 to 2 years old were the highest-risk group due to accidental access to dangerous substances, and males were more likely to experience these injuries (26).

Additionally, LSC dysfunction has been described as a potential precursor to, or subtype of, LSCD. LSC dysfunction has been described previously in a series of patients in whom corneal epithelial changes were reversed by medical therapy (3). Kim, et al. proposed that LSC dysfunction can occur due to disruption of the limbal microenvironment, or niche, in which the limbal stem cells may have the potential to be rehabilitated (3). This may occur most commonly with chronic contact lens use or BAK toxicity. If left untreated, these patients can develop persistent damage to the limbal niche, and subsequent irreversible LSCD.

## 3 Diagnosis – symptoms, slit lamp examination, and clinical staging

LSCD symptoms can vary from asymptomatic to severe. Photophobia, redness, and foreign body sensation are common (27). Other symptoms may include tearing, dryness or general ocular discomfort and pain. This wide variety of symptoms make LSCD’s presentation non-specific, which necessitates clinical evaluation, including with slit lamp biomicroscopy.

**Figure 1:**
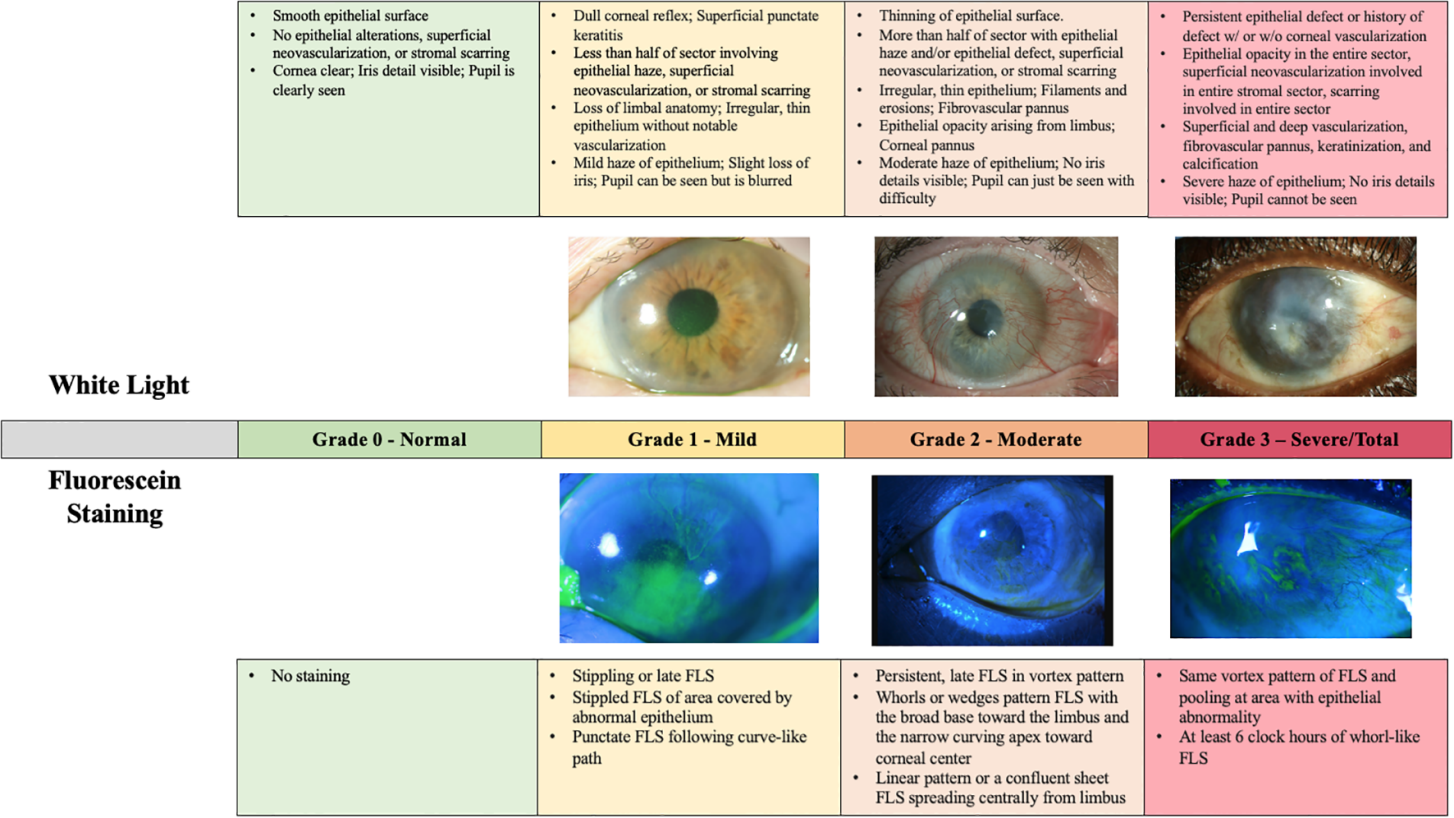
LSCD grading per international consensus guidelines (1). Each stage is subdivided by the degree of limbal involvement. All photographs were captured by Joshua Hou MD at the University of Minnesota. For the images of both Stage 1A and Stage 3 LSCD, the white light and fluorescein photos are matched from the same patient. For the images of Stage 2B LSCD, photos from two different patients were used.

In 2019, the Cornea Society’s Limbal Stem Cell Working Group established a clear definition of LSCD and criteria for diagnosis, classification, and staging. The Working Group first divided LSCD into partial and total LSCD (1). Total LCSD was characterized as loss of all LSCs that causes full conjunctivalization of the cornea. LSCD staging based on clinical presentation was defined as the following: Stage 1 is disease where the central 5mm of the cornea maintains normal corneal epithelium; Stage 2 is disease where the central 5mm of the cornea is affected; Stage 3 is disease where the entire corneal surface is affected (1). These stages are sub-divided further based on the extent of limbal involvement: A) less than 50% limbal involvement, B) greater than 50% but less than 100% limbal involvement, and C) 100% limbal involvement (**Figure 1**) (1).

**Figure 2:**
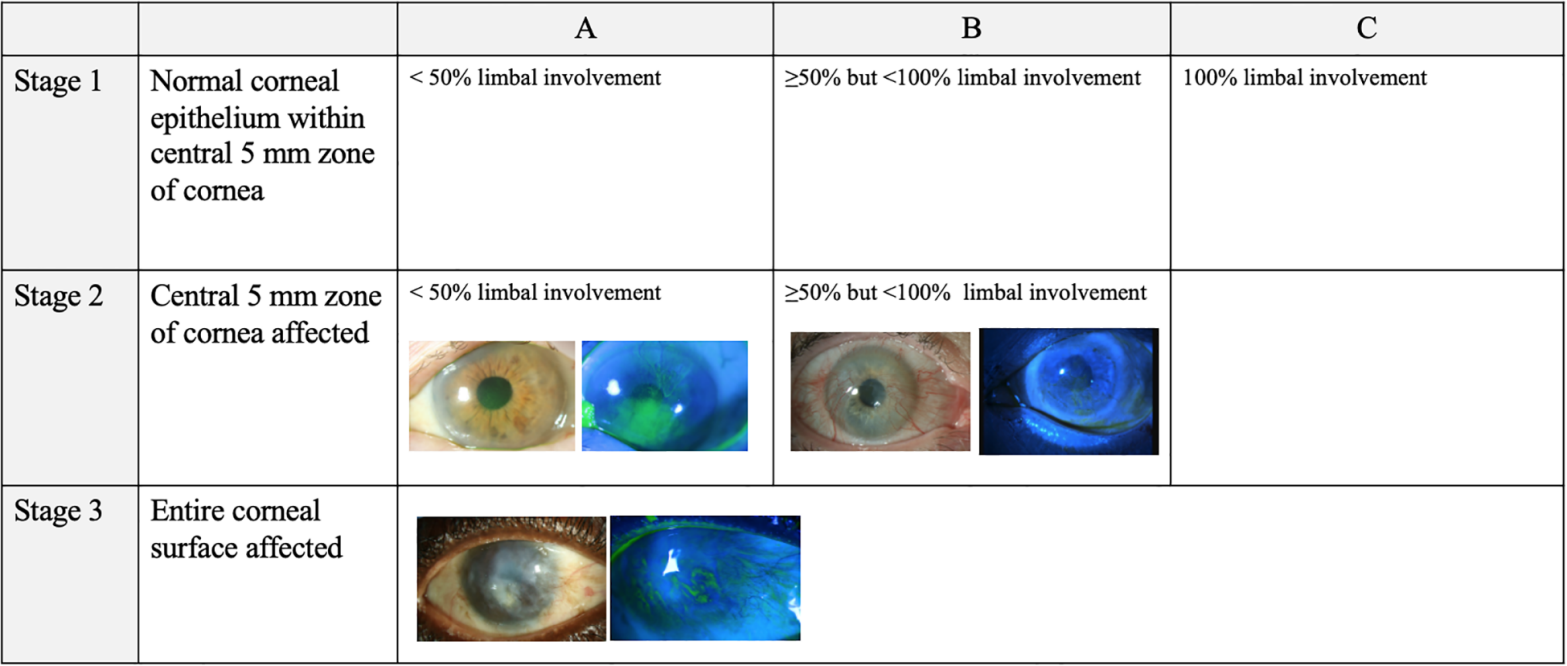
LSCD grading according to Le et al., 2018 by slit lamp microscopy with white light versus fluorescein staining. This figure provides descriptive explanations of the various possible findings in mild, moderate, and severe LSCD in both white light and fluorescein staining. The figure text was primarily adapted from Le et al., 2018 (27) and supplemented by other papers (1, 26, 29). Of note, there exists a degree of overlap between the mild, moderate, and severe grades based on the Le et al., 2018 grading scale. All photographs were captured by Joshua Hou MD at the University of Minnesota. For the images of both mild and severe LSCD, the white light and fluorescein photos are matched from the same patient. For the images of moderate LSCD, photos from two different patients were used.

Findings on slit lamp examination included late vortex or punctate fluorescein staining, reduced epithelial transparency, epithelial irregularities and recurrent epithelial defects, neovascularization, absence of palisades of Vogt, an opaque or scarred cornea, and an inflamed ocular surface (28). **Figure 2** is a previously published descriptive grading scale adapted from Le, et al.’s review on LSCD. This grading scale is useful in determining disease severity but is not widely used clinically (28).

Slit lamp examination remains an important clinical method for detection of LSCD, especially given its wide availability. In 2020, Le, et al. outlined 105 studies where LSCD was diagnosed based on only clinical examination in 62.9% of eyes (2). A confirmatory diagnostic test was used in 25.8% of eyes, with impression cytology (IC) used in 24.2% of eyes, *in vivo* confocal microscopy (IVCM) used in 0.7% of eyes, and a combination IVCM and IC used in 0.9% of eyes (2). The remaining 11.3% of eyes diagnosed with LSCD had no diagnostic criteria provided in their studies (2). In patients with LSC dysfunction, slit lamp examination is also critical, with the predominant finding being a whorl-like or wave-like epitheliopathy (3). Pathologically, these patients often have a continuous sheet of single clones of late staining epithelium that follow a whorl-like pattern, which may represent a form of metaplasia. Other studies have demonstrated the pathologic presence of goblet cells on the cornea in cases of LSCD resulting from trauma or inflammation (29).

While slit lamp examination is often the initial method to detect LSCD, the use of confirmatory measures is important to review.

## 4 Diagnostics modalities

### 4.1 Imaging modalities

Imaging modalities have become widely popular for the diagnosis of LSCD and offer objective means of grading LSCD. Objective measures of LSCD can also help in cases where the diagnosis or staging are unclear.

### 4.2 *In vivo* confocal microscopy

*In vivo* confocal microscopy (IVCM) is a noninvasive imaging technology that can visualize microstructures in the cornea, limbus, and conjunctiva at a cellular level. It has lateral resolution of 1 µm and axial resolution of 4µm. It can also be used in healthy and abnormal eyes, offering an excellent comparison in case of unilateral disease (28). Multiple studies have shown loss of palisades of Vogt, epithelial phenotype changes, neovascularization of the cornea, and loss of sub-basal nerves in LSCD (30). Microstructural changes on IVCM have also been demonstrated in LSCD, even in early stages. Epithelial cells in patients with LSCD are less distinct and have more prominent nuclei. In severe LSCD, epithelial cells can appear metaplastic with severely reduced cell density. There is also severe reduction in epithelial thickness centrally in LSCD, and more severe thinning is associated with worse LSCD (28).

Goblet cells have also been used as a diagnostic marker in LSCD. The presence of goblet cells on corneal IVCM can confirm the diagnosis of LSCD (31). However, detection of goblet cells on IVCM is challenging and highly dependent on the skill of examiner. IVCM has low sensitivity for detecting goblet cells due to the small area that is scanned, so multiple regions of the cornea may need to be analyzed. Furthermore, goblet cells have been reported to have both hypo (32) and hyper-reflective cytoplasm (31), which can lead to diagnostic challenges for examiners unfamiliar with their appearance on IVCM.

A decrease in basal cell density with increase in the size of cells has been reported in LSCD. These changes also correspond with disease severity (33). A basal cell density of <7930 cell/mm^2^ is sufficient to diagnose LSCD (95.5% sensitivity and 100% specificity) (33–35). In very severe cases there in an increase in the hyperreflectivity of the cell nuclei. There can also be an increase in dendritic cells in the stroma, which can assist in diagnosing partial LSCD, where there are areas of clinically normal epithelium (34).

Corneal nerve changes observed in IVCM can also help with the diagnosis of LSCD. A decrease in the sub-basal plexus of nerves is associated with the severity of disease; as severity worsens, there is eventually complete nerve drop out. A decrease in corneal nerve branch length, increase in branching angulation, and increase in tortuosity is associated with LSCD (33, 36). A corneal nerve density of < 53 nerves/mm^2^ has an 87% sensitivity and 91.7% specificity for diagnosing LSCD (33).

### 4.3 Anterior segment optical coherence tomography

AS-OCT is another non-invasive tool that can be used to diagnose LSCD with repeatable results. Like IVCM, a decrease in corneal and limbal epithelial thickness in AS-OCT can be seen in eyes with LSCD. In LSCD, 20-30% epithelial thinning has been reported, compared to other epitheliopathies, where thinning can be up to 10% (6, 32, 33). This distinction between degree of epithelial thinning makes AS-OCT a helpful tool in supporting a diagnosis of LSCD.

Newer parameters, such as the mean of the central epithelial thickness and thickness measured at 1mm on either side of central, have been proposed by Liang, et al. as a way to diagnose LSCD (37). Values <46.6um were considered diagnostic for LSCD with a sensitivity of 61.7% and specificity of 100% (37).

AS-OCT can also be used for *in vivo* visualization of the palisades of Vogt. IVCM can also visualize the palisades of Vogt but requires more technical expertise. Furthermore, one can also appreciate significant epithelial thinning when the palisades are absent, which can be another clue to the presence of LSCD (38). Epithelial reflectivity is another method that can be used to detect LSCD (39). A ratio of 1.29 or greater between epithelial and stromal reflectivity can be indicative (40).

### 4.4 Impression cytology

Impression cytology can provide objective evidence of LSCD and has historically been considered the gold standard for diagnosis of LSCD. However, impression cytology has some disadvantages; importantly, it cannot determine disease severity, and false negatives can occur if involved areas are not sampled. The test involves sampling the superficial epithelial cells on the ocular surface. These cells are obtained by applying nitrocellulose or cellulose acetate filter paper to the corneal surface (34). As the filter paper is removed, adherent superficial epithelial cells are removed with the paper. Repeated sampling in a particular area can help access deeper layers.

Immunohistochemistry is then performed on the sampled cells to diagnose LSCD. This is typically done by staining goblet cells. Goblet cells in the corneal epithelium indicate LSCD; however, the quantity of goblet cells does not necessarily correlate with disease severity. The sensitivity of the test is also highly related to the quality of the specimen taken and may be influenced by the filter paper and the staining methods used. A lack of goblet cells on impression cytology in patients with LSCD may be due to sampling error, such as in partial LSCD, or cell loss during the sampling procedure. Therefore, the absence of goblet cells does not rule out LSCD (34). Due to these issues, there is a risk for false negative results with impression cytology (34).

Several histopathological stains are used to determine the present of LSCD. Stains for goblet cell mucin, including hematoxylin and eosin, Giemsa, and Periodic acid-Schiff, can help detect goblet cell invasion over the cornea, which is a key feature of LSCD. Other immunohistochemical markers for conjunctival epithelium or goblet cells can also aid in diagnosis. Cytokeratin 12 is a marker of mature corneal epithelium. In contrast, cytokeratin 7,13, and 19 are specific markers expressed in conjunctival epithelial cells and goblet cells. Muc5ac is another mucin stain for identifying goblet cells (34). Using such markers along with mucin stains can help aid in the diagnosis of LSCD and reduce the risk of false negatives.

Conjunctivalization of the cornea is considered the hallmark of LSCD. Typically, normal corneal epithelial cells adhere tightly to the basement membrane, making impression cytology challenging. In LSCD, conjunctival cells on the cornea can desquamate more freely, thus having an abundance of cells is further evidence of LCSD (34).

### 4.5 Optical coherence tomography-angiography

OCT angiography (OCT-A) is a non-invasive test that evolved is similar to spectral domain-OCT (SD-OCT). It is typically used to assess retinal vasculature by picking up signals from red blood cell movement from sequential scans in the same location. It has also been used in the anterior segment to image corneal neovascularization and limbal vasculature, but its utility in LSCD requires further investigation (41). One study found that corneal vascular extension (COVE) and corneal vascular thickness (COVT) strongly correlate with disease severity and best corrected vision in patients with LSCD (41). Still, more work is needed to establish a definitive role of OCT-A in the evaluation of LSCD.

## 5 Discussion

Recent years have seen significant advancements in the diagnosis of LSCD. Efforts to establish an international consensus on LSCD diagnostic criteria and staging have helped standardize our definition of LSCD. While slit lamp examination remains the primary method for diagnosing LSCD in most clinical settings, advanced diagnostic tools, such as IVCM, AS-OCT, impression cytology, and OCT-A, have improved our ability to confirm the diagnosis. Though the detection of conjunctival epithelium and goblet cells on impression cytology remains the gold standard for LSCD diagnosis, the use of IVCM, AS-OCT, and OCT-A are important non-invasive tools for supporting the diagnostic approach to LSCD.

Such tools are not only important for diagnosing LSCD, but also may help guide treatment. For example, three-dimensional mapping by either OCT or IVCM may help identify deep limbal lacunae and determine if there are residual limbal epithelial cells present.

Furthermore, diagnosis and staging of LSCD may be important for determining whether medical or surgical therapy is appropriate. For example, if LSCD is identified, medical therapy can immediately be initiated to optimize the ocular surface including frequent lubrication, serum tears, or therapeutic contact lenses (18). Those with confirmed LSCD may also be candidates for surgical treatments including corneal epithelial removal with or without amniotic membrane transplantation or one of various forms of limbal stem cell transplantation, depending on unilateral versus bilateral involvement as well as disease severity. The staging and diagnosis of LSCD is therefore critical to determining which treatment options may be indicated.

There is an ongoing need for improved diagnostic modalities for LSCD. Quantifiable markers such as basal cell density, sub-basal nerve density, and epithelial thickness are useful for diagnosing LSCD but often require a skilled operator to reliably obtain them. Further improvements in image resolution, field of view, and image analysis software will be important for expanding the use of ICVM and AS-OCT in the diagnosis of LSCD. Having clear diagnostic criteria for LSCD and being able to diagnose it early with accurate severity grading is critical to ensuring patients with LSCD receive appropriate treatment. The current and ongoing research on the diagnosis, grading, and management of LSCD will help achieve this goal.
